# Structural Characterization and Abundance of Sialylated Milk Oligosaccharides in Holstein Cows during Early Lactation

**DOI:** 10.3390/foods13162484

**Published:** 2024-08-07

**Authors:** Lisa Isernhagen, Christina E. Galuska, Andreas Vernunft, Sebastian P. Galuska

**Affiliations:** Research Institute for Farm Animal Biology (FBN), Wilhelm-Stahl-Allee 2, 18196 Dummerstorf, Germany; isernhagen@fbn-dummerstorf.de (L.I.); vernunft@fbn-dummerstorf.de (A.V.)

**Keywords:** sialic acids, milk oligosaccharides, sialyllactose, bovine milk, colostrum, lactation

## Abstract

Among other bioactive molecules, milk contains high amounts of sialylated milk oligosaccharides (MOs) that influence numerous processes in the offspring. For instance, sialylated MOs inhibit the invasion of pathogens and positively influence the gut microbiome to support the optimal development of the offspring. For these reasons, sialylated MOs are also used in infant formula as well as food supplements and are potential therapeutic substances for humans and animals. Because of the high interest in sialylated bovine MOs (bMOs), we used several analytical approaches, such as gas and liquid chromatography in combination with mass spectrometry, to investigate in detail the profile of sialylated bMOs in the milk of Holstein Friesian cows during early lactation. Most of the 40 MOs identified in this study were sialylated, and a rapid decrease in all detected sialylated bMOs took place during the first day of lactation. Remarkably, we observed a high variance within the sialylation level during the first two days after calving. Therefore, our results suggest that the content of sialylated MOs might be an additional quality marker for the bioactivity of colostrum and transitional milk to ensure its optimized application for the production of milk replacer and food supplements.

## 1. Introduction

The immune system of vertebrates is highly influenced by glycans [1,2,3,4,5,6]. There are diverse possibilities in which such oligo- and polysaccharides modulate the immune response. For instance, as a part of the glycocalyx, which surrounds all living cells, glycans attached to membrane proteins and lipids can function as an identification template. In this case, heterogeneous carbohydrate structures are recognized by different glycan-binding proteins of immune cells, allowing a distinction between endogenous and exogenous cells [6]. Such glycan-dependent inactivation or activation mechanisms of the immune system are not only mediated by the glycans of glycoproteins and glycolipids but also by freestanding glycan structures. In mammals, milk oligosaccharides (MOs) belong to these unattached immunomodulatory glycans and are important to support the optimal development of the offspring [7,8,9,10].

MOs are linear or branched carbohydrate structures. In human milk, the initial base is the disaccharide lactose that consists of a glucose (Glc) and a galactose (Gal) residue and can be further elongated by the addition of N-acetylglucosamine (GlcNAc), Gal, and fucose (Fuc) residues and the sialic acid N-acetylneuraminic acid (Neu5Ac) [11]. Human MOs (hMOs) are the third major group of biomolecules in human milk. With a concentration of ~1.3 g/100 mL, mature human milk contains more MOs than proteins (~1 g/100 mL), and only lactose and fat are present in higher concentrations, at ~6.8 and ~3.9 g/100 mL, respectively [12].

To date, more than 200 different hMOs [13], which are involved in numerous physiological processes, have been described. For example, MOs positively influence the gut microbiome [13] since they are metabolic substrates for colonic bacteria [14,15]. Scientists were able to identify differences between the composition of bacteria from the feces of children who were breastfed compared to those who were bottle-fed and revealed that MOs play an important role in the observed effects on the microbiome [16]. Moreover, MOs alter the glycocalyx composition of intestinal epithelial cells to prevent the binding of pathogenic microorganisms [17]. For these reasons, MOs have been increasingly described as prebiotics and are essential for the optimal development and health of newborns [7,18]. In addition, MOs can directly affect immune cells. For instance, sialylated hMOs reduce the expression of IL-1β and IL-6, which are proinflammatory cytokines [17]. Another study reported that sialylated hMOs induced the release of IFN-γ and IL-10 [19]. The authors concluded that acidic hMOs may regulate allergen-specific immune responses by suppressing Th-2-type responses in atopy-prone individuals [19]. In addition, free MOs have further essential functions to support the innate immune system. For many pathogens, including viruses, bacteria, and protozoan parasites, the first step of infection is the attachment to epithelial cells, and frequently, glycans of the glycocalyx are the major binding targets. Interestingly, there are many structural similarities between the glycan motifs on the endogenous cell surface and MOs. Thus, the adhesion of pathogens is inhibited, when the binding pockets of pathogenic receptors are blocked by MOs. Examples of such pathogens are norovirus, rotavirus, the protozoan parasite *Entamoeba histolytica*, *Salmonella* spp., *H. pylori*, and *C. sakazakii* [17,20,21,22,23,24].

With antibiotic resistance becoming an increasingly common problem in animal husbandry and humans, carbohydrates, such as MOs, may represent a natural alternative to prevent or treat infections of the respiratory system and the gut [11,17,25,26]. Therefore, bovine colostrum has been used in veterinary practice as well as in complementary therapy in humans for many years [27], and the infant formula industry uses bovine milk as an established base for the production of infant formula [28]. Bovine milk is also sold as a dried powder or utilized as a functional ingredient in cheeses, nutritional bars, milk powder beverages, yogurts, and other products to promote health [27,29]. Another important aspect is the use of bovine colostrum in animal husbandry in the form of a milk replacer for calves after separation from the dam [30].

Most of the positive impacts of MOs are mediated by fucosylated and sialylated structures. However, in contrast to hMOs, fucosylation is low to absent in bovine MOs (bMOs) [31]. Thus, sialylated structures represent the key MOs in bovine milk. Intriguingly, the sialic acids Neu5Ac and N-glycolylneuraminic acid (Neu5Gc) are used to elongate bMOs, whereas Neu5Ac represents the only sialic acid that can be synthesized in humans because of a mutation in the CMAH gene, which encodes the enzyme that hydroxylates Neu5Ac to Neu5Gc [32]. However, Neu5Gc from food, such as cow’s milk and meat, can be incorporated into human glycans and found in human tissues after voluntary ingestion [33]. Remarkably, humans have anti-Neu5Gc antibodies. Thus, Neu5Gc attached to glycans of the glycocalyx induces a xeno-autoantigen reaction since Neu5Gc is recognized as “nonself” by the human immune system but as “self” by human glycosylation pathways [34]. As a consequence, anti-Neu5Gc antibodies are associated with accelerated inflammation in atherosclerosis and other inflammatory diseases [35]. One study from 2008 even suggested that local inflammation as a result of the metabolic accumulation of Neu5Gc contributes to the formation of diet-related carcinomas [36]. Thus, the Neu5Gc content in food is relevant for a healthy diet and should be low. This is important for considering bovine milk for human and infant consumption.

Since bMOs are not only important for supporting the optimal development of cow offspring but are also frequently used to produce infant formula and are potential therapeutic biomolecules in humans and animals, it is important to determine the structure and proportion of sialylated MOs to estimate the positive and negative bioactivity of the sialylated MO fraction in bovine milk [37]. For this reason, we characterized the composition of sialylated MOs during the first days of lactation in detail using different chromatographic methods in combination with mass spectrometry.

## 2. Materials and Methods

### 2.1. Sample Collection

The milk samples of German Holstein dairy cows (Holstein Friesian cows; Bos taurus taurus) were obtained from the experimental cattle facility of the Research Institute for Farm Animal Biology (FBN). Milk was collected at five different time points directly after calving (d0, first milking, colostrum), on days 1 and 2 postpartum (p.p.) (transitional milk), and approximately on days 7 and 30 p.p. (mature milk). After collection, the samples were shock frozen, transported under frozen conditions, and stored at −20 °C until use.

### 2.2. Extraction of Sialylated Milk Oligosaccharides

To extract sialylated MOs, we skimmed the milk samples using double centrifugation at 13,000 rpm for 30 min at 4 °C. After that, solid phase extraction was performed using a PGC cartridge with a 25 mg column bed weight (HyperSep™ Hypercarb™ SPE Cartridges, Thermo Fisher Scientific) according to the protocol of Blank et al. [38]. The flow rate was regulated using a vacuum pump with a reduced pressure of approximately 800 mbar to ensure dropwise flow. After equilibration, 300 µL of the 1:10 diluted sample was loaded onto the cartridge. After washing and eluting, the extracted MOs were stored at −20 °C until further processing.

### 2.3. Quantification of Sialic Acids

The sialic acids Neu5Ac and Neu5Gc were quantified using a 1,2-diamino-4,5-methylenedioxybenzene (DMB)-based strategy using a Shimadzu HPLC system equipped with a fluorescence detector and a protocol based on previous studies [39,40,41,42]. We used this method for the analysis of both whole milk and the extracted bMOs fraction. First, hydrolysis had to be performed to release the sialic acid residues. Different amounts of Neu5Ac (Carbosynth Limited, Compton, UK) and Neu5Gc (Sigma–Aldrich, Taufkirchen, Germany) were used to calculate a calibration line. As an internal standard, we used ketodeoxynonulosonic acid (KDN; Sigma–Aldrich, Taufkirchen, Germany) in all standards and samples since we detected no KDN in bovine milk. For the analysis of sialic acids in milk, 2 µL of the milk samples were diluted in 398 µL of ddH_2_O; 100 µL of this dilution was used for hydrolysis after drying in a vacuum concentrator (SpeedVac, Uniequip Univapo 150H). For the analysis of sialic acids in the extracted MO fractions, the samples were used after drying. Hydrolysis was conducted with 100 µL of 0.4 N TFA at 80 °C for 4 h. After centrifugation at 13,000 rpm for 30 min and drying, the samples were then stored at −20 °C until further derivatization with 40 µL of ddH_2_O and 40 µL of DMB reagent (1.22 mg of DMB dissolved in 1 mL of buffer, consisting of 18 mM sodium hydrosulfites, 1 M mercaptoethanol, and 40 mM TFA) at 55 °C for 2 h. The reaction was stopped by adding 20 µL of 0.2 N sodium hydroxide (NaOH). A Superspher 100 RP-18 decapped LiChroCART 250-2 column was used for separation with ACN:methanol:ddH_2_O at a ratio of 4:4:92 + 0.1% TFA as mobile phase A. Mobile phase B consisted of the same compounds at a ratio of 45:45:10 + 0.1% TFA. The flow rate was 0.25 mL/min. The following gradient was applied: 0 min, 100% mobile phase A; 2 min, 100% mobile phase A; 25 min, 98% mobile phase A; 35 min, 95% mobile phase A; 40 min, 50% mobile phase A; 45 min, 100% mobile phase B; 50 min, 100% mobile phase B; 51 min, 100% mobile phase A; and 60 min, 100% mobile phase A. The temperature of the column oven was set to 45 °C, and the samples were stored in a coolable autosampler at 4 °C. DMB derivatives were detected using an RF-10A XL fluorescence detector with an excitation wavelength of 372 nm and an emission wavelength of 456 nm.

### 2.4. LC-MS-MS Analysis of Extracted Milk Oligosaccharides

Extracted MOs were dried using a SpeedVac and then taken up in 100 µL of 80% acetonitrile (ACN, Merck, Darmstadt, Germany). A ThermoFisher Vanquish UHPLC equipped with an AccuCore-150-Amide-HILIC and corresponding guard column was used for the separation of the MOs. The mobile phases consisted of A: 50% ACN with 10 mM ammonium acetate (Merck, Darmstadt, Germany) and 0.1% acetic acid and B: 90% ACN with 10 mM ammonium acetate (Merck, Darmstadt, Germany) and 0.1% acetic acid. The following gradient was used with a flow rate of 0.4 mL/min: 0 min, 35% mobile phase A; 27 min, 80% mobile phase A; 28 min, 95% mobile phase A; 33 min, 95% mobile phase A; 33.1 min, 35% mobile phase A; and 45 min, 35% mobile phase A. The eluted MOs were detected with HESI–MS/MS in negative ionization mode using a Qexactive plus mass spectrometer (Thermo Fisher, Dreieich, Germany). For the detection of fucosyllactose (FL), we used the positive ion mode. The mass range was set to *m*/*z* 300–2800 with a resolution of 70,000 for full MS and 17500 for data-dependent MS/MS. For the structural analysis, MS and MS^2^ spectra as well as the GlycoWorkbench 2 software were used [43]. The peak areas of the total ion chromatograms (TICs) and EICs were determined using FreeStyle Software (version 1.8, Thermo Fisher Scientific, Dreieich, Germany).

### 2.5. Quantification of Fucose

Fucose (Fuc), one of the building blocks of MOs, was quantified with gas chromatography (GC) after hydrolysis of the oligosaccharides and subsequent reduction and peracetylation of Fuc [44,45]. The applied procedure is based on Brunton et al. [46]. For calibration, combinations of different monosaccharide standards, including Fuc (Sigma, Taufkirchen, Germany), were used. As an internal standard, a defined amount of xylose (Xyl, Sigma, Taufkirchen, Germany) was added to all extracted samples and standards. Between the following reaction steps, the samples were dried under nitrogen flow (approx. 1 bar) in a heating block (Bioblock Scientific) at a temperature of approximately 35 °C. Hydrolysis of the MOs took place in 500 µL of 4 N trifluoroacetic acid (TFA, Roth, Karlsruhe, Germany) for 4 h at 100 °C. After drying, the reduction was performed with 500 µL of 1% sodium borohydride solution (Sigma–Aldrich, Taufkirchen, Germany) overnight at room temperature. Then, 100 µL of 2 N acetic acid (Roth, Karlsruhe, Germany) was added after reduction. The samples were then washed with a 4-fold mixture of 2.5 mL of methanol (MeOH) (MS grade, Merck, Darmstadt, Germany) and 1% acetic acid with redrying in between. For acetylation, the samples were dissolved in 100 µL of pyridine (Sigma–Aldrich, Taufkirchen, Germany) and 400 µL of acetic anhydride (Sigma–Aldrich, Taufkirchen, Germany) and incubated overnight at room temperature in the dark. For the final extraction of the peracetylated Fuc, the dried samples were extracted using 4 mL of dichloromethane (DCM, Merck, Darmstadt, Germany) and 2.5 mL of ddH_2_O. After shaking for 5 min and centrifuging at 3000 rpm for 3 min, the aqueous phase was discarded using annealed Pasteur pipettes and a vacuum pump. This procedure was repeated two more times before the samples were dried and stored at 4 °C until further processing. For the analysis, the peracetylated samples and standards were dissolved in 100 µL of DCM and transferred to glass vials (Agilent, Waldbronn, Germany). For the analysis, samples and standards were injected onto an Agilent Technologies 7890A GC series set operating with electron ionization and coupled with an Agilent Technologies 5975C series MS with a triple-quadrupole detector. For separation of the analytes, a HP-5MS column with Helium as carrier gas with a constant flow of 1 mL/min was used. The transfer line was heated to 280 °C, and the inlet was heated to 260 °C with a septum purge flow of 3 mL/min and a purge flow to a split vent of 50 mL/min at 0.5 min. The following temperature gradient was used: 0 min, 80 °C; 1 min, 80 °C; 11 min, 180 °C; 21 min, 180 °C; 57.667 min, 290 °C; followed by a 5 min postrun at 130 °C. Selected ion monitoring (SIM) mode was used for detection with the resolution set to 1.5 and an electron multiplier (EM) voltage of 600. For the generation of the extracted ion chromatograms, we used the following mass/charge ratios: Fuc, 217 and 231; Xyl, 145 and 217.

### 2.6. Statistical Analysis

The statistical analysis and visualization were performed using the website BioRender.com and the “Graphs” function, which use different packages of the programming language R (version 4.2.2) to perform all the statistical analyses. In this study, all the results were statistically analyzed using one-way ANOVA and Tukey’s multiple comparison test. The significance levels are indicated as follows: *: *p* < 0.05, **: *p* < 0.01, ***: *p* < 0.001, ****: *p* < 0.0001.

## 3. Results and Discussion

### 3.1. Sialylation of MOs in Holstein Milk during Lactation

Earlier studies described a rapid decrease om Neu5Ac in bovine milk within the first days of lactation [26,47,48]. Nakamura et al. reported a sialic acid concentration of 1700 ng/µL in bovine colostrum, which decreased to 520 ng/µL during the first 24 h and further to 150 ng/µL on day 7 after parturition [26]. The data of our recent study supported the described course of the Neu5Ac concentration in Holstein cow milk with Neu5Ac values between 1079 and 2728 ng/µL, averaging approximately 1980 ng/µL on the day of calving, which rapidly decreased to approximately 200 ng/µL (10%) in mature bovine milk [47]. It has to be mentioned that the amounts of Neu5Ac detected in whole milk samples are from glycoproteins, glycolipids, MOs, and free sialic acids.

**Figure 1:**
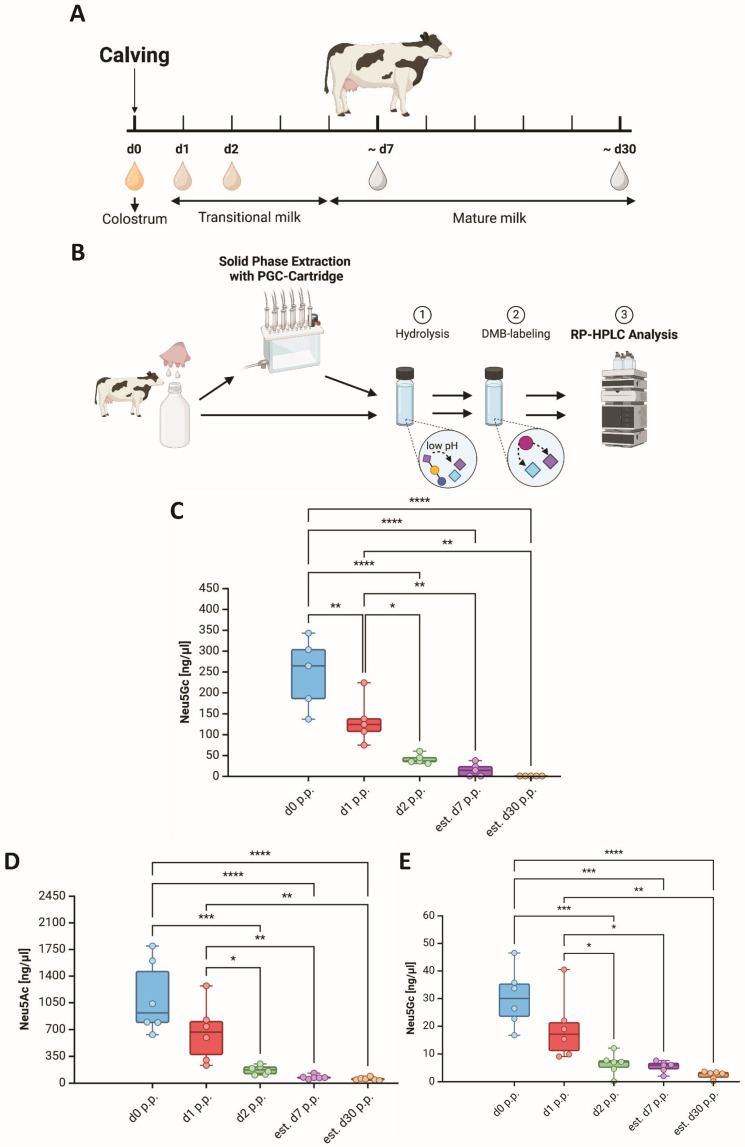
Quantification of Neu5Gc in milk from Holstein Friesian cows. (**A**) Sampling timeline for milk collection. The time ranges of colostrum, transitional milk, and mature milk were specified on the basis of Silva et al. [49]. Created with BioRender.com. (**B**) Scheme of the DMB-RP-HPLC strategy for sialic acid quantification. Sialic acid residues in milk as well as in the extracted bMOs fraction were released by hydrolysis and subsequently labeled with DMB for fluorescence detection using an RP-HPLC system equipped with a fluorescence detector. Created with BioRender.com. (**C**) Box and whisker plots (median; min to max) showing the Neu5Gc values during early lactation (n = 6 animals) as well as the values for Neu5Ac (**D**) and Neu5Gc (**E**) in the extracted bMOs fraction (n = 5 animals). The statistical analysis and graphs were generated using BioRender.com. Significant differences are denoted as follows: *: *p* < 0.05, **: *p* < 0.01, ***: *p* < 0.001, ****: *p* < 0.0001.

With the same sample setup (Figure 1A), the Neu5Gc concentrations were also calculated. For the RP-HPLC analysis, the sialic acid residues were released using TFA hydrolysis and subsequently labeled with DMB (Figure 1B). On the day of parturition, the Neu5Gc values varied between 136 and 342 ng/µL, with an average concentration of 246 ng/µL (Figure 1C). On day 7 p.p., an average of 24 ng/µL indicates a 14-fold decrease of Neu5Gc during the first week of parturition. A quantitative analysis of Neu5Gc for all samples was only possible until day 2 p.p. since most of the values were under the limit of detection on day 7 and 30 p.p. (3 of the 5). As already described for Neu5Ac, we also observed great variations in the Neu5Gc values in bovine colostrum but little to no variation in mature milk samples. These differences might be the result of metabolic stress, which can be compensated to varying degrees by the cows [47].

To quantify the sialic acid content on bMOs during early lactation, MOs were extracted using PGC cartridges (Figure 1B). It should be noted that only sialylated MOs are quantitatively extracted using solid-phase extraction, while uncharged MOs have to consist of more than three neutral monosaccharides to be extracted [38]. Nevertheless, lactose and neutral MOs smaller than a tetramer are also partially extracted with PGC cartridges. However, these neutral MOs do not influence the quantification of Neu5Ac and Neu5Gc using the outlined DMB-RP-HPLC application. The calculated Neu5Ac concentrations attached to MOs varied between 628 and 1791 ng/µL, averaging approximately 1100 ng/µL on the day of calving and decreasing to approximately 50 ng/µL on day 30 p.p. (Figure 1D). Thus, the Neu5Ac values decreased by a factor of 22. As already observed during the analysis of sialic acids in whole milk, there is great individual variation in MOs from colostrum and lower variation in MOs from mature milk. Similar to Neu5Ac, Neu5Gc shows wide variety on day 0 p.p. with values of 17–46 ng/µL, with an average of 30 ng/µL, whereas the dispersion decreases with ongoing lactation, similar to the course of Neu5Ac. On day 30 p.p., we quantified only ~3 ng/µL Neu5Gc, which corresponds to a 10-fold decrease during early lactation (Figure 1E).

The observed values at 2 p.p. are in line with those of Martín-Sosa et al. [50]. The authors reported values for sialic acids attached to MOs of approximately 231 mg/kg in bovine transitional milk on day 2 p.p. and only approximately 38 mg/kg on day 7 p.p. [50]. They described an insignificant decrease between day 7 p.p. and mature milk 3 months after parturition. However, they did not differentiate between Neu5Gc and Neu5Ac. Their values are thus the sum of both. Assuming that 1 L of milk weighs 1 kg, the sialic acid concentration of Martín-Sosa et al. corresponds to 231 ng/µL on day 2 p.p. and 38 ng/µL on day 7 p.p. When adding up the values for Neu5Ac and Neu5Gc for our analysis, we quantitated a sialic acid concentration of 175 ng/µL for day 2 p.p. and approximately 80 ng/µL for day 7 p.p. Thus, the values for day 2 p.p. are in the same range, whereas on day 7 p.p., we observed approximately 2 times higher concentrations in comparison to the reported quantities by Martín-Sosa et al. An explanation for this discrepancy might be the difference in the analyzed bovine breed. Martín et al. used milk samples from Spanish-Brown cows and not from Holstein Friesian cows. Other studies have shown that MO profiles depend on the breed and origin of the cattle [51,52,53,54] as well as on different feeding strategies [55,56].

### 3.2. Distribution of Sialylated MOs during Lactation

**Figure 2:**
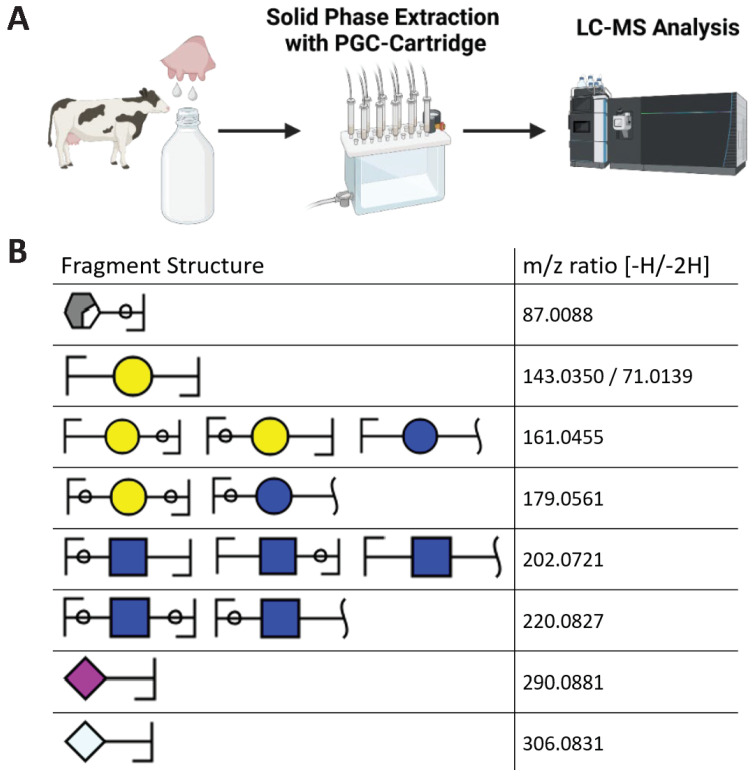
Analysis of bMO distribution using LC-MS. (**A**) Scheme of bMO analysis using LC-MS. After bMO enrichment using PGC cartridges, the resulting bMOs were analyzed using HILIC-HESI-MS/(MS). Created with BioRender.com. (**B**) The displayed MS fragments of bMOs were used for the identification of bMOs. For the manual verification process, at least one of those fragments had to be found in the corresponding MS^2^ spectrum to identify the detected bMO. For the sialylated bMOs, the most significant fragment was always the cleaved sialic acid residues (Neu5Ac or Neu5Gc). The bMO structures were computed with GlycoWorkbench 2 [43].

Since with most extraction strategies, contamination with glycoconjugates, such as glycopeptides, cannot be completely excluded, the structure and abundance of bovine sialylated MOs during early lactation were analyzed in detail with a UHPLC system equipped with an AccuCore-150-Amide-HILIC column that was directly coupled to a tandem MS system (Figure 2A). For the optimal detection of the sialylated MOs, we used the negative ion mode. Using the applied HILIC column, the oligosaccharides are separated according to their polarity.

First, the complete MO profile was determined. To this end, we extracted the specific deprotonated ions of 54 known MOs from the obtained MS data. When we observed the expected mass of an MO, the MS^2^ spectra were examined for fragments corresponding to the structural composition (Figure 2B). Typical fragments of sialylated MOs match the loss of the terminal sialic acid residue. In the case of Neu5Ac, a deprotonated pseudomolecular ion [M-H]^−^ at 290.0881 *m*/*z* is an indicator, whereas for Neu5Gc, an ion at 306.0831 *m*/*z* is detectable. For Hex, deprotonated pseudomolecular fragment ions [M-H]^−^ at 143.0350 *m*/*z* ([M-2H]^2−^: 71.0139 *m*/*z*), 161.0455 *m*/*z*, and 179.0561 *m*/*z* are typical. In addition, a detected mass of 87.0088 *m*/*z* resembles a cross-ring fragmentation of the 6-carbon ring of a Hex. For the MOs containing a HexNAc residue, we observed fragment ions at 202.0721 and 220.0827 *m*/*z*.

**Figure 3:**
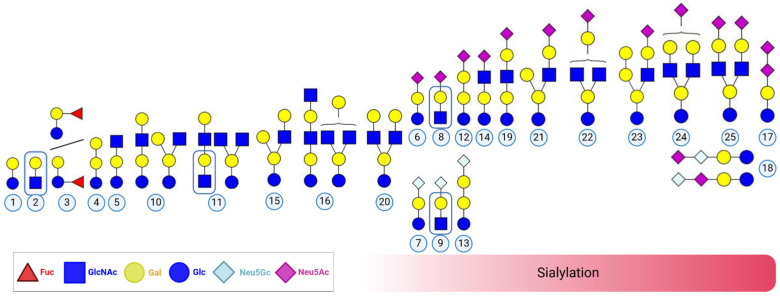
Proposed structures of the detected disaccharides lactose and lactosamine in addition to bMOs in Holstein Friesian cows during early lactation. For some bMOs, multiple linkages and compositions have been reported. Due to the lack of specific linkage analysis and for clarity purposes, not all possible isomers are visualized. The bMO structures were designed with GlycoWorkbench 2 [43] and assembled using BioRender.com. Each structure is given the corresponding number used in this study, which is also described in Table 1.

**Figure 4:**
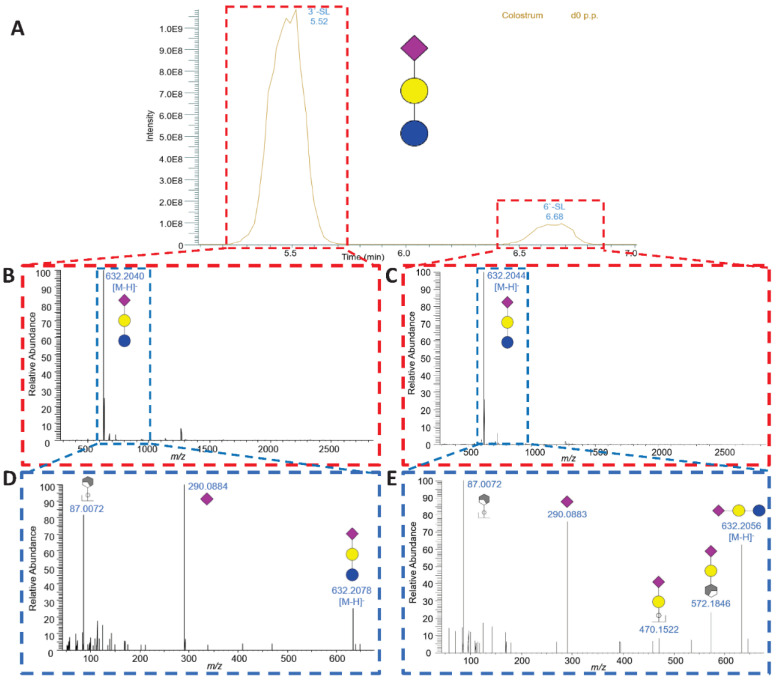
LC-MS analysis of sialyllactose (SL). (**A**) Extracted ion chromatogram (EIC) of SLs shown for representative samples on the day of calving. (**B**) MS spectrum at 5.46 min showing the deprotonated molecular ion [M-H]^−^ of SL with its corresponding MS/MS spectrum (**D**) showing two MO fragments as a cross-ring fragment of a Hex with 87.0072 *m*/*z* and a detached Neu5Ac at 290.0884 *m*/*z*. (**C**) MS spectrum at 6.68 min showing the deprotonated molecular ion [M-H]^−^ of SL with its corresponding MS/MS spectrum (**E**) showing fragments at 87.0072 and 290.0883 *m*/*z*, as well as Neu5Ac attached to a Hex (470.1522 *m*/*z*) and Neu5Ac attached to a Hex with an additional cross-ring fragment of another Hex (572.1846 *m*/*z*). The bMO structures were designed with GlycoWorkbench 2 [43].

Based on the MS and MS^2^ spectra, we identified 40 different bMOs, including 26 different *m*/*z* ratios and 15 isomers, in the milk of Holstein Friesian cows throughout early lactation (Figure 3 and Table 1). The milk glycans ranged from disaccharides, identified as lactose (#1) and lactosamine (#2), to more complex bMOs consisting of up to 8 monosaccharides, such as bMO #25 (Hex_4_HexNAc_2_Neu5Ac_2_). This bMO represents one of the 29 identified sialylated bMOs. Only 9 of those 29 acidic bMOs were sialylated with Neu5Gc, which is in line with the detected difference in the concentration of both sialic acids. One of the bMOs containing Neu5Gc is the trisaccharide Neu5Gc-lactose (NGL, #7, Hex_2_Neu5Gc, [M-H]^−^: 648.1993) (Supplemental Figure S1). In addition, two further variants of sialyllactose (SL) were observed to contain an α2,3- or α2,6-linked Neu5Ac residue (3′- and 6′-SL #6) (Figure 4). Using SL standards, retention times of 5.5 and 6.7 min were determined for 3′- and 6′-SL, respectively (Supplemental Figure S2). The EIC demonstrated that 3′-SL is the dominant form of all SL variants. Remarkably, 3′-SL is recognized by *E. coli* K99 and can be used to prevent diarrhea in piglets [57,58]. This approach was also successfully tested for oral application for protecting colostrum-deprived newborn calves [59], which further illustrates the importance of sialylated bMOs for the immune system and explains why 3′- and 6′-SL have been added to infant formula in more than 30 countries for more than 30 years [60,61,62,63]. In addition, the highly pathogenic avian influenza (HPAI) A(H5N1) virus in dairy cattle seems to use the α2,3-gal motif as binding structure in mammary gland tissues [64,65]. Thus, the bMO 3′-SL might be an efficient inhibitor against the invasion of HPAI H5N1 to epithelial cells in mammary gland of Holstein Friesian cows.

**Figure 5:**
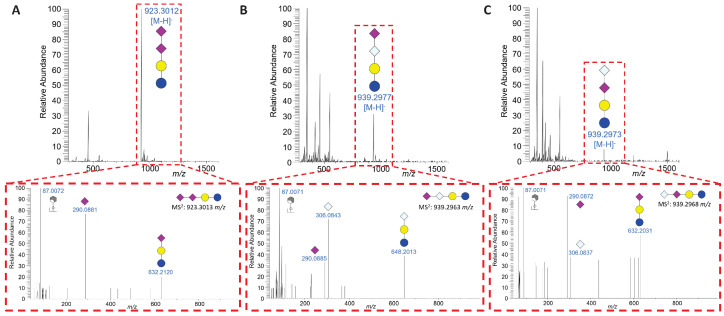
LC-MS analysis of DSL. (**A**) MS spectrum at 10.99 min showing the deprotonated molecular ion for DSL containing a Neu5Ac dimer attached to the lactose core and the corresponding MS^2^ spectrum for DSL at this time. The MS^2^ spectrum shows three fragments, two of which are already mentioned for SL in Figure 4 (87.0072 and 290.0881 *m*/*z*), and one larger fragment is SL with 632.2120 *m*/*z*, validating the linear composition of DSL. (**B**) MS spectrum at 11.51 min showing the deprotonated molecular ion for heterogenic DSL (hDSL) consisting of Neu5Ac as well as Neu5Gc attached to the lactose core and the corresponding MS^2^ spectrum for hDSL at this time. The MS^2^ spectrum shows four fragments, two of which were already mentioned for SL in Figure 4 (87.0071 and 290.0885 *m*/*z*), as well as one larger fragment, NGL, with 648.2013 *m*/*z*, validating the linear composition of hDSL as NGL with an additional Neu5Ac attached. (**C**) The MS spectrum at 12.69 min displays the deprotonated molecular ion for hDSL and the corresponding MS^2^ spectrum for hDSL at this time. The MS^2^ spectrum shows four fragments, three of which were already mentioned for hDSL (87.0071, 290.0872, and 306.0837 *m*/*z*), as well as SL with 632.2031 *m*/*z*, validating the linear composition of the second hDSL as SL with an additional Neu5Gc attached. The bMO structures were designed with GlycoWorkbench 2 [43].

Two of the acidic bMOs contain a Neu5Ac dimer. One of these is named disialyllactose (DSL, #17) (Figure 5A). This MO consists of a lactose core and an α2,8-linked Neu5Ac dimer. In addition to DSL, lactose with two different sialic acid dimers consisting of Neu5Ac(α2,8)Neu5Gc (Figure 5B) or Neu5Gc(α2,8)Neu5Ac (Figure 5C), which we named “heterogenic disialyllactose” (hDSL, #18), were observed. The obtained MS spectra demonstrate that Neu5Gc and Neu5Ac can terminate the heterogenic sialic acid dimers on DSL, which has been unknown to date. In addition, we detected the lactonized forms of DSL and hDSL (Supplemental Figure S1). The lactone rings are formed by an internal esterification of the carboxyl group of the terminal sialic acid residue and the hydroxyl group at C9 of the internal sialic acid residue [73,74,75]. Remarkably, all three forms of DSL have not yet been identified in human milk, and the biological impact on offspring and humans is largely unknown.

The only fucosylated bMO detected in this study was fucosyllactose (FL, #3, [M+Na]^+^: 511.1633 *m*/*z*; Hex_2_Fuc_1_; Supplemental Figure S3). In terms of fucosylated bMOs, contrary results have been published. For example, although the presence of Fuc(α1-3)Gal(β1-4)GlcNAc in bovine colostrum was shown by Saito et al. [76], Tao et al. could not confirm this, as they could not detect any fucosylated free bMOs in bovine milk [72]. However, Mariño et al. confirmed two other fucosylated glycans, 2′-FL and a structure consisting of GalNAc(α1,3)[Fuc(α1,2)]Gal(β1,4)Glc, in bovine colostrum from Holstein Friesian cattle [77]. To verify the presence of fucosylated structures using an additional approach, we quantified Fuc with a GC–MS system equipped with an HP-5MS column. To this end, the extracted MOs were hydrolyzed, and the released Fuc residues were subsequently reduced and peracetylated. As expected, we detected only low levels of Fuc (Supplemental Figure S4). In the MO fraction from colostrum, Fuc was present at concentrations between 31.06 and 67.46 ng/µL, whereas in MOs from mature milk on day 30 p.p., Fuc residues were not detectable in most of the samples (2 of 6). Thus, the obtained GC–MS results support the occurrence of FL, which we detected with LC-MS. Considering this, fucosylation is indeed present in bMOs but only in small amounts. The low fucosylation status of bovine milk is also in line with the findings of Shi et al. [31]. However, we detected no sialylated bMOs in Holstein milk containing fucose residues in this study.

Sulfo-lactose was also detected in the samples (Supplemental Figure S5), as it has been described in rat milk [78,79] as well as bovine milk [80]. Sulfated MOs have long been known to be present in the milk of different mammals. As early as 1961, sulfated SL was found in rat mammary gland tissue [81]. In 1985, a study also mentioned the presence of sulfated SL in human milk as an explanation for the simultaneous delivery of two essential nutrients, sulfate and calcium, via milk [78]. They speculated that sulfated MOs are hydrolyzed in the gut of neonates and absorbed as inorganic sulfate without the precipitation of calcium sulfate in the milk. Only a few years ago, the concentration of sulfated hMOs were shown to correlate with the suppression of two major cytokines, IL-10 and IL-13, and therefore affect the immune system [82]. However, we only observed sulfo-lactose and no sulfation of sialylated MOs. Additional well-known MOs, which were detectable in the extracted samples, were lacto-N-tetraose (LNT, #10) and sialyl-lacto-N-tetraose (LST, #19) (Supplemental Figure S1).

**Figure 6:**
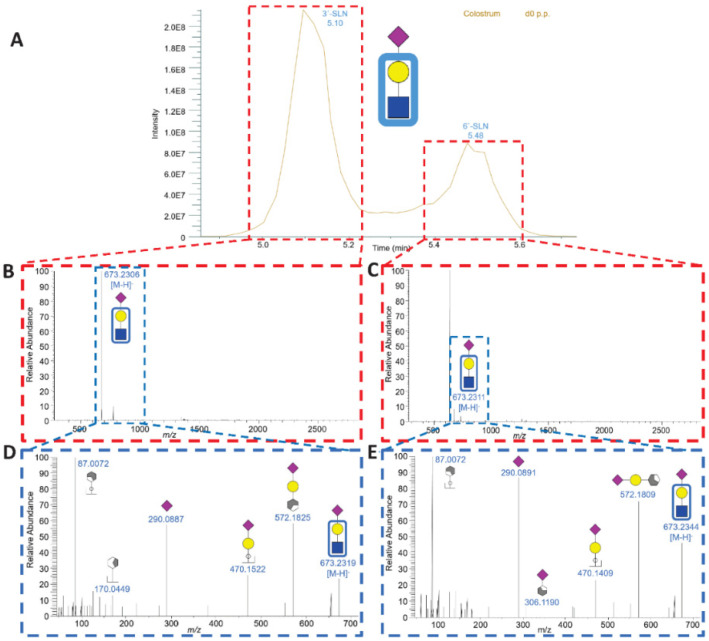
LC-MS analysis of sialyllactosamine (SLN). (**A**) Extracted ion chromatogram (EIC) of SLN shown for a representative sample on the day of calving. (**B**) MS spectrum at 5.10 min showing the deprotonated molecular ion 673.2306 *m*/*z* of SLN with the corresponding MS^2^ spectrum, (**D**) which displays the same fragments as those displayed for SL in Figure 5, with an additional fragment of another cross-ring fragment of Hex at 170.0449 *m*/*z*. (**C**) MS spectrum at 5.50 min showing the deprotonated molecular ion 673.2311 *m*/*z* of SLN with the corresponding MS^2^ spectrum, (**E**) which shows the same fragments as noted at 5.10 min. Additionally, there is another fragment of Neu5Ac attached to a cross-ring fragment of a Hex (306.1190 *m*/*z*), which indicates the α2,6-linkage of Neu5Ac. The bMO structures were computed with GlycoWorkbench 2 [43].

Among the 40 bMOs, the majority contains a lactose core. However, four bMOs are based on a lactosamine core (#2, #8, #9, and #11) (Supplemental Figure S1 and Figure 6). Lactosamine is an additional core structure for bMOs [12,13,58,72,83], whereas the initial base of all hMOs is lactose [84,85]. Two of these structures are sialylated lactosamine (#8, #9) containing either Neu5Gc (NGLN) or Neu5Ac (SLN). It was possible to distinguish between 3′- and 6′-SLN. In MS^2^, the deprotonated pseudomolecular fragment ion [M-H]^−^ at 306 *m*/*z* indicates an α2-6-linked Neu5Ac residue at 5.5 min, while its absence indicates the presence of an α2,3-linked Neu5Ac residue at 5.1 min [79] (Figure 6). The biological function of these sialylated structures is currently unknown. However, it can be speculated that the binding of comparable pathogens to epithelial cells is inhibited, as described for SL, since recognition is commonly mediated by the Neu5Ac-Gal motif. Interestingly, SLN is detectable in the blood of infants [86]. The authors assumed that SLN in infant blood is a degradation product of more complex hMOs. Since such complex MOs are atypical in cattle, NGLN and SLN might be directly synthesized by endogenous glycosyltransferases.

**Figure 7:**
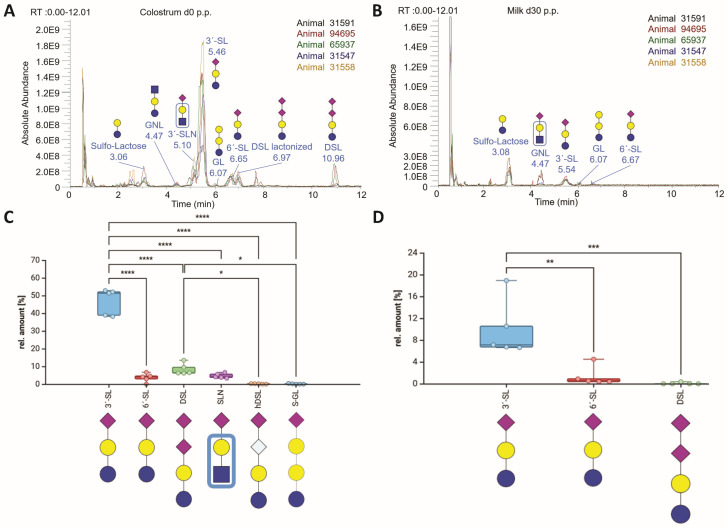
Distribution of bMOs during lactation. (**A**) The total ion chromatography (TIC) from the colostrum samples on day 0 p.p. of the analyzed Holstein cattle were overlaid using FreeStyle software (Thermo Fisher). The MO peaks were labeled with the name and retention time of the base peak. (**B**) The TIC from the colostrum samples on day 30 p.p. of the analyzed Holstein cattle were overlaid using FreeStyle software. The MO peaks were labeled with the name and retention time of the base peak. The ratios of the peak areas of the sialylated bMOs were determined (n = 5 animals), and box and whisker plots (median; min to max) are shown for (**C**) day 0 and (**D**) day 30 p.p. Only sialylated bMOs with peak areas greater than 0.1% were included. The statistical analysis and graphs were generated using BioRender.com. Significant differences are denoted as follows: *: *p* < 0.05, **: *p* < 0.01, ***: *p* < 0.001, ****: *p* < 0.0001. The bMO structures were designed with GlycoWorkbench 2 [43].

To determine the abundance of the detected sialylated MOs, the TICs were analyzed, which revealed that on the day of calving, 3′-SL was the dominant MO in colostrum, followed by DSL, 6′-SL, SLN, and hDSL with terminal Neu5Ac (Figure 7A,B). For DSL, the lactonized derivative was also visible in the TIC at approximately 7 min. For all other sialylated MOs, the signal intensity was too low in the TIC, and we detected only minor signals for the neutral MOs GNL and GL in addition to sulfo-lactose. Several articles have reported that the predominant bMOs in colostrum are 3′-SL, 6′-SL, SLN, and DSL, which account for approximately 70% of the total MOs present [26,31,51,80].

Moreover, the signals obtained for the sialylated MOs differed greatly between the individuals. For example, the TIC peak for 3′-SL varied between an absolute abundance of approximately 5 × 10^8^ and nearly 1.9 × 10^9^. We also observed this strong variance in the outlined sialic acid quantification using RP-HPLC. These results are in line with the research of Liu et al. in 2017, where remarkable individual variations were observed [68]. The individual differences in the sialylated MOs might be the result of metabolic stress during the transition phase, which might be compensated to varying degrees by the cows [47]. As shown for the sialic acid levels, the signals for the sialylated structures rapidly decreased during lactation (Figure 7C,D).

**Figure 8:**
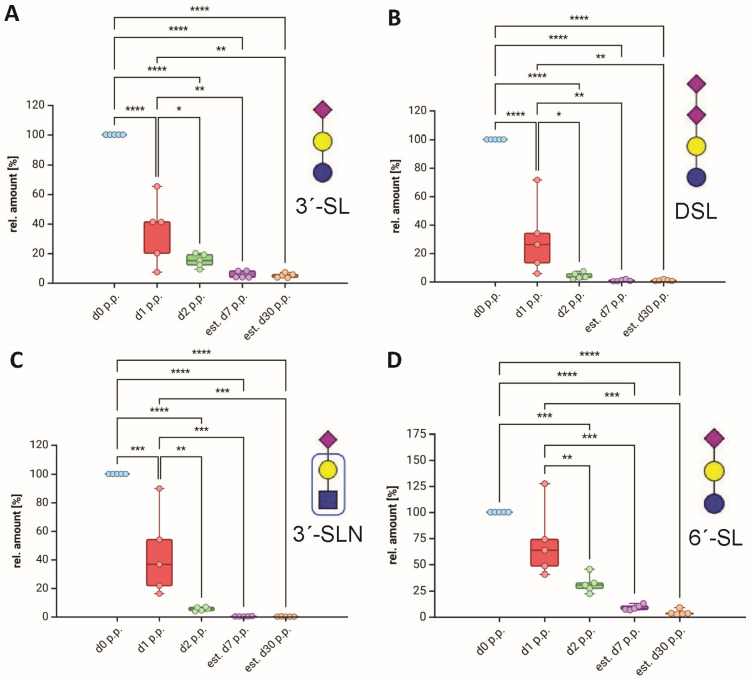
Abundance of the major sialylated bMOs during early lactation. The EICs were used to calculate the ratios of the peak areas of the most abundant sialylated bMOs. Box and whisker plots (median; min to max) are shown for (**A**) 3′-SL, (**B**) DSL, (**C**) 3′SLN, and (**D**) 6′-SL (n = 5 animals). Statistical analysis and graphs were generated using BioRender.com. Significant differences are denoted as follows: *: *p* < 0.05, **: *p* < 0.01, ***: *p* < 0.001, ****: *p* < 0.0001. The bMO structures were designed with GlycoWorkbench 2 [43].

To determine the relative amounts of all sialylated bMOs during lactation, the EICs of these glycans were used, which is called “label-free” relative quantification [13]. In comparison to the TICs, the EICs have the advantage that only specific ions are analyzed, whereas the TIC is the sum of all ions at a certain retention time. Thus, other potential ions at the retention site influence the peak area in the TIC. As expected, the relative amount of all sialylated structures rapidly decreased (Figure 8 and Supplemental Figure S1). This trend is particularly evident in the two dominant MO fractions, 3′-SL and DSL (Figure 8). One day after calving, the values of 3′-SL and DSL decreased by more than 50%. On day 2 p.p., less than 20% of 3′-SL and less than 10% of DSL were still detectable. This trend also applies to most other sialylated MOs. However, for some MOs, such as SGL, the relative amounts only decreased noticeably two days after calving (Supplemental Figure S1). Nevertheless, on day 30 p.p., only very small amounts of all sialylated bMOs remained (Figure 8 and Supplemental Figure S1). Whereas in previous studies, only the courses of two to six sialylated bMOs were described [28,51,80,87,88,89,90], in our study, the abundance of 26 sialylated bMOs was monitored during lactation (Supplemental Figure S6). The results demonstrate that the rapid decrease of sialylated biomolecules, such as lactoferrin and IgG [87,91], during early lactation applies also to all sialylated bMOs.

## 4. Conclusions

In summary, our lab successfully identified 29 sialylated bMOs (including three lactonized diasialylated MOs) and 11 neutral bMOs in the milk of Holstein cows. Thus, the majority of those bMOs were sialylated, which showed a rapid decrease during early lactation. In addition to the great differences in the abundance of bMOs depending on the time of lactation, there was also a high variation between the different cows during the first days of lactation. Thus, it is important to determine the content of sialylated MOs to estimate the bMO-dependent bioactivity of colostrum and transitional milk to ensure an optimal calf development. The same applies when colostrum is used to produce food supplements or for potential complementary therapies in humans. Moreover, bovine colostrum is most likely the most efficient source for the purification of sialylated MOs since the chemical synthesis is still very expensive. Thus, the sialylation status may be useful for quality control and as a biomarker for determining the pathogen inhibition functionalities and immunomodulatory impact of milk mediated by sialylated MOs, such as SL.

## Figures and Tables

**Table 1:** MOs and disaccharides found in Holstein Friesian cows during early lactation. The *m*/*z* values for the deprotonated molecular ions [M-H]^−^ and/or [M-2H]^2−^ (for FL: sodium adduct [M+Na]^+^) of the detected glycans are listed with the assigned number and retention time (RT) as well as the name used in this study. The composition is indicated according to Hex_HexNAc_Fuc_Neu5Ac_Neu5Gc. This table is based on the following references: [31,50,66,67,68,69,70,71,72].

#	Composition	No. of Building Blocks	Name	Category	[M-H]^−^	[M-2H]^2−^	[M+Na]^+^	RT
1	2_0_0_0_0	2	Lactose	neutral	341.1089			3.1
1	2_0_0_0_0	2	Sulfo-lactose	neutral	421.0657			3.1
2	1_1_0_0_0	2	Lactosamine	neutral	382.1355			2.2
3	2_0_1_0_0	3	Fucosyllactose (FL)	neutral			511.1633	4.5
4	3_0_0_0_0	3	Galactosyllactose (GL)	neutral	503.1618			6.1
5	2_1_0_0_0	3	Galactosaminyllactose (GNL)	neutral	544.1883			4.7
6	2_0_0_1_0	3	3′-Sialyllactose (SL)	acidic	632.2044			5.5
6	2_0_0_1_0	3	6′-Sialyllactose (SL)	acidic	632.2044			6.7
7	2_0_0_0_1	3	3′-Neu5Gc-lactose (NGL)	acidic	648.1993			7.0
7	2_0_0_0_1	3	6′-Neu5Gc-lactose (NGL)	acidic	648.1993			8.2
8	1_1_0_1_0	3	3′-Sialyllactosamine (SLN)	acidic	673.2309			5.1
8	1_1_0_1_0	3	6′-Sialyllactosamine (SLN)	acidic	673.2309			5.5
9	1_1_0_0_1	3	3′-Neu5GC-lactosamine (NGLN)	acidic	689.2258			6.3
9	1_1_0_0_1	3	6′-Neu5GC-lactosamine (NGLN)	acidic	689.2258			6.8
10	3_1_0_0_0	4	Lacto-N-tetraose (LNT)	neutral	706.2411			7.6
11	2_2_0_0_0	4		neutral	747.2677			5.7
12	3_0_0_1_0	4	Sialyl-galactosyllactose (S-GL)	acidic	794.2572			8.2
12	3_0_0_1_0	4	Sialyl-galactosyllactose (S-GL)	acidic	794.2572			8.6
12	3_0_0_1_0	4	Sialyl-galactosyllactose (S-GL)	acidic	794.2572			8.9
12	3_0_0_1_0	4	Sialyl-galactosyllactose (S-GL)	acidic	794.2572			9.2
13	3_0_0_0_1	4	Neu5Gc-galactosyllactose (NG-GL)	acidic	810.2521			10.3
14	2_1_0_1_0	4		acidic	835.2837			7.8
15	4_1_0_0_0	5		neutral	868.2940	433.6433		11.3
16	3_2_0_0_0	5		neutral	909.3205	454.1566		10.1
17	2_0_0_2_0	4	Disialyllactose (DSL)	acidic	923.2998	461.1462		11.0
17	2_0_0_2_0	4	Disialyllactose (DSL) lactonized	acidic	905.2892	452.1410		7.0
18	2_0_0_1_1	4	“Neu5Ac-NGL (hDSL)”	acidic	939.2947	469.1437		11.5
18	2_0_0_1_1	4	“Neu5Gc-SL (hDSL)”	acidic	939.2947	469.1437		12.7
18	2_0_0_1_1	4	“Neu5Ac-NGL (hDSL)” lactonized	acidic	921.2841	460.1384		8.2
18	2_0_0_1_1	4	“Neu5Gc-SL (hDSL)” lactonized	acidic	921.2841	460.1384		8.5
19	3_1_0_1_0	5	3′-Sialyl-lacto-N-tetraose (LST)	acidic	997.3365	498.1646		10.5
19	3_1_0_1_0	5	6′-Sialyl-lacto-N-tetraose (LST)	acidic	997.3365	498.1646		12.1
20	4_2_0_0_0	6	Lacto-N-hexaose (LNH)	neutral	1071.3733	535.1830		13.2
21	4_1_0_1_0	6		acidic	1159.3894	579.1910		13.9
21	4_1_0_1_0	6		acidic	1159.3894	579.1910		15.3
22	3_2_0_1_0	6		acidic	1200.4159	599.7043		12.7
22	3_2_0_1_0	6		acidic	1200.4159	599.7043		14.0
23	5_1_0_1_0	7		acidic	1321.4422	660.2175		16.8
24	4_2_0_1_0	7		acidic	1362.4687	680.7307		17.1
25	4_2_0_2_0	8		acidic	1653.5642	826.2784		20.3

## Data Availability

Data are included in the article and Supplementary Materials; further inquiries can be directed to the corresponding author.

## Supplementary Materials

The following supporting information can be downloaded at: https://www.mdpi.com/article/10.3390/foods13162484/s1, Supplemental Figure S1: LC-MS data of all detected sialylated bMOs in Holstein Friesian cows during early lactation; Supplemental Figure S2: Extracted ion chromatogram of 3′-SL (black) and 6′-SL (red) standards showing the respective retention time of both isomers; Supplemental Figure S3: LC-MS analysis of fucosyllactose (FL); Supplemental Figure S4: Quantification of fucose (Fuc); Supplemental Figure S5: LC-MS analysis of sulfated lactose; Supplemental Figure S6: Sampling timeline in previous studies and the outlined analysis.
